# Older men in motion: bodies, masculinities, and redefinition of identity

**DOI:** 10.3389/fpsyg.2025.1556790

**Published:** 2025-02-17

**Authors:** Gustavo González-Calvo, Alfonso García-Monge, André Ramalho, Faten Hamdi, Pedro Duarte-Mendes

**Affiliations:** ^1^Departamento de Didáctica de la Expresión Musical, Plástica y Corporal, Research Group Embodied Education, Universidad de Valladolid, Valladolid, Spain; ^2^Department of Sports and Well Being, Polytechnic Institute of Castelo Branco, Castelo Branco, Portugal; ^3^Sport Physical Activity and Health Research and Innovation Center, SPRINT, Santarém, Portugal; ^4^High Institute of Sport and Physical Education of Kef, University of Jendouba, Le Kef, Tunisia

**Keywords:** active aging, emotional well-being, affect, body awareness, physical exercise

## Abstract

**Introduction:**

This study explores the intersection of physical exercise, masculinity, and aging in older men, examining how these elements shape identity and bodily experiences. Aging is often framed through deficit-based narratives that emphasize decline; however, this research seeks to reframe aging as a process of adaptation and opportunity. By investigating how physical activity contributes to identity reconstruction, this study aims to provide insights into the broader social and structural factors influencing older men’s engagement in exercise.

**Methods:**

The study employs a qualitative research design, utilizing semi-structured interviews with 12 men aged 65–76. A narrative thematic analysis was conducted to examine participants’ experiences, concerns, and expectations regarding physical activity. This approach allowed for an in-depth exploration of how exercise intersects with notions of masculinity and aging, as well as the barriers and facilitators that shape participation in physical activities.

**Results:**

The analysis identified three key themes: (1) the transformative role of exercise in fostering emotional well-being and bodily awareness, (2) the renegotiation of traditional masculinity through engagement in non-traditional physical activities, and (3) the social connections cultivated through group-based exercise programs. Additionally, the study highlights social and structural barriers, such as limited access to facilities and economic constraints, which hinder participation in physical activity.

**Discussion:**

Findings underscore the significance of physical activity not only for maintaining health but also as a space for identity reconstruction and resistance to conventional narratives of aging. The study suggests that exercise serves as a means of empowerment, allowing older men to navigate aging in ways that challenge societal expectations. Moreover, the identification of structural barriers emphasizes the need for inclusive public health policies and targeted interventions that promote active aging. By reframing aging as a dynamic process of adaptation rather than mere decline, this research contributes to a broader understanding of how older adults experience and engage with physical activity.

## 1 Introduction

Aging, traditionally associated with loss, decline, and dependency, has long been framed within a deficit model that underscores physical frailty, cognitive deterioration, and social withdrawal. This perspective has contributed to cultural narratives that often marginalize older adults, portraying them as passive recipients of care rather than active agents in shaping their lives. However, contemporary approaches to aging challenge these reductive views, emphasizing its dynamic and transformative potential. Recent scholarship, including the work of Dionigi et al. (2013), has shed light on how older adults negotiate the aging process, focusing on their capacity for adaptation, identity renegotiation, and meaningful engagement with the world around them.

For older men, this transformative view of aging intersects with the complexities of masculinity. Masculinity, as a socially constructed identity, evolves over the life course, shaped by cultural expectations, generational norms, and personal experiences. Physical activity, in this context, emerges not only as a tool for maintaining health and well-being but also as a significant arena for older men to reconstruct their understanding of masculinity. By engaging in physical activity, older men can challenge traditional gender norms that emphasize dominance, physical strength, and competitiveness, instead embracing practices that prioritize emotional well-being, social connection, and self-care. This shift reflects a broader societal trend toward reimagining masculinity as a more fluid and inclusive construct (Gough and Robertson, 2010).

Despite these opportunities for transformation, older men’s experiences with physical activity are not free from tension. The intersection of aging and masculinity often brings to the forefront societal expectations that perpetuate restrictive stereotypes. Cultural ideals of masculinity frequently valorize youth, vitality, and physical prowess, creating pressures that can exacerbate feelings of inadequacy as men age. Simultaneously, the physical changes associated with aging—such as reduced mobility, slower recovery times, and increased vulnerability to injury—pose challenges to older men’s ability to engage in physical activities in the ways they once did. These tensions highlight the need to explore how older men navigate the dual influences of societal expectations and the realities of aging bodies (Clarke et al., 2020; Fernández-Ballesteros et al., 2017; Phoenix et al., 2005).

In this context, physical activity offers a unique space for older men to negotiate these complexities. Exercise becomes more than a practice for physical maintenance; it acts as a medium for exploring identity, fostering resilience, and cultivating social bonds. Through activities such as walking groups, yoga classes, or resistance training, older men can engage in embodied practices that challenge traditional narratives of decline. Instead, they can embrace aging as a process of growth, adaptation, and self-discovery. Furthermore, group physical activities provide opportunities for older men to combat social isolation, a common issue in retirement, by fostering inclusive and supportive environments (Bredland et al., 2018; Mulcahy, 2012; Klinenberg, 2018).

This study seeks to examine these dynamics by focusing on the particularities of masculinity and its influence on perceptions of the body, exercise, and social interaction in later life. Using a qualitative approach, it explores how older men perceive and experience physical activity, how they confront the bodily changes associated with aging, and how these practices affect their sense of identity and belonging. By delving into participants’ narratives, this research illuminates the ways in which older men navigate the intersections of gender and aging, offering insights into how physical activity can serve as a space of resistance, adaptation, and personal reaffirmation (Clarke et al., 2020; Sparkes, 2015).

The significance of this study lies in its potential to challenge deficit-based narratives of aging, providing a more nuanced understanding of how older men experience and embody their identities. By focusing on the interplay between the body, movement, and masculinity, this research contributes to broader discussions on active aging and the transformative potential of physical activity. It highlights how older men’s engagement in exercise not only enhances their physical and emotional well-being but also offers a platform for renegotiating societal norms, fostering resilience, and cultivating meaningful connections. These findings hold implications for public health initiatives, social policy, and cultural discourse, underscoring the need to create inclusive and accessible environments that empower older adults to thrive in later life.

Through its exploration of these themes, this study aims to expand the understanding of aging as a dynamic and possibility-filled process, demonstrating how the intersections of gender, embodiment, and social context shape the lived experiences of older men. By doing so, it seeks to contribute to a richer and more inclusive narrative of aging, one that recognizes the diverse ways in which individuals navigate and redefine their identities in later life.

### 1.1 Corporeality, masculinity, and aging: dynamic and transformative perspectives

Aging in Western societies has traditionally been conceptualized through a model of decline, focusing on the loss of abilities, dependency, and physical and cognitive frailty (Dionigi et al., 2013). This approach has reinforced cultural narratives that associate aging with limitations and social disconnection, often obscuring the diversity of experiences during this stage of life. However, contemporary perspectives have challenged these one-dimensional views, proposing a more complex and dynamic understanding of the body as an entity in constant transformation, capable of generating meaning and capabilities throughout the life cycle (Garrett, 2004). This framework is grounded in theories of corporeality, which emphasize the relationship between bodies, individual experiences, and social contexts.

Corporeality provides a comprehensive lens to understand the body not merely as a physical entity, but as a space for cognitive, emotional, and social interaction. According to Merleau-Ponty (2012), the body is not simply an object but a medium through which the world is experienced and imbued with meaning. In the context of aging, this perspective allows for an examination of how older men perceive physical changes in their bodies and how these changes impact their identities and capabilities. This study reveals that despite the limitations associated with aging, physical activity can serve as a transformative resource that enables older men to reaffirm their autonomy, adapt to bodily transformations, and maintain an active connection with their environment (Bredland et al., 2018; Clarke et al., 2020; Hydén, 1997; Phoenix et al., 2005; Sparkes, 2015). Exercise, in this sense, emerges as a means of negotiating the aging process, reconfiguring both perceived capacities and personal identity.

Masculinity, understood as a social construction, plays a central role in this negotiation. Cultural norms and gender stereotypes have historically defined masculine ideals around attributes such as strength, resilience, and physical dominance (Clarke et al., 2020; Clarke, 2012; Gough and Robertson, 2010; Sparkes, 2015). These traditional conceptions of masculinity have influenced how older men confront aging, particularly in the realm of physical exercise. However, recent experiences documented in this study reveal that many older men are challenging these norms by engaging in non-traditional physical activities such as yoga or Pilates, which have historically been perceived as feminine practices (Phoenix et al., 2005; Sloan et al., 2010). These practices not only promote physical well-being but also offer an opportunity to reconfigure masculinity in aging, moving away from traditional ideals and adopting a more holistic approach to health and well-being (Gough and Robertson, 2010).

Group physical activities also play a key role in promoting active and socially integrated aging. This study highlights how group dynamics can overcome initial barriers associated with gender norms, creating inclusive spaces where older men find mutual support and acceptance (Mulcahy, 2012; Phoenix and Sparkes, 2008, 2010). These group activities, in addition to facilitating social interaction, also strengthen connections with the natural environment, allowing participants to enjoy outdoor activities that foster a renewed sense of autonomy and vitality (Garrett, 2024). The social dimension of exercise, therefore, not only contributes to physical well-being but also reinforces community ties and a sense of belonging.

In this context, physical activity becomes a space for identity renegotiation where older men can challenge dominant narratives of decline and vulnerability (Clarke et al., 2020; Phoenix and Sparkes, 2006, 2008; Sparkes, 2015). By engaging in physical practices that prioritize health, enjoyment, and social connection, participants reconfigure their corporeality, transforming perceived limitations into opportunities to explore new ways of being and inhabiting their bodies. This process, deeply interwoven with notions of masculinity, underscores how bodily practices can serve as a vehicle for reconfiguring not only personal identity but also the social dynamics surrounding aging (Sparkes, 2015).

In summary, theories of corporeality and active aging propose a transformative vision of male aging, emphasizing the capacity of older bodies to adapt, resist, and reinterpret social expectations (Bredland et al., 2018; Phoenix and Sparkes, 2006; Sparkes, 2015). Far from being an inevitable period of decline, aging emerges as a stage of creative potential and profound connection with oneself and others (Dionigi et al., 2013). These psychosocial transformations help explain why, in many contexts, aging is often experienced as a period of greater subjective well-being (Fernández-Ballesteros et al., 2017; Steptoe et al., 2015). In this sense, physical activity stands out as a crucial tool for challenging limiting social constructions, fostering autonomy, and exploring new ways of living and experiencing old age.

## 2 Methodology

### 2.1 Participants and training program

This study involved 12 men aged 65–76 years who participated in a physical activity program aimed at preventing cognitive decline. The program, known as “Cogn-Mover” (in an effort to integrate the concept of cognition, movement, and emotion) emphasized the integration of cognition, movement, and social interaction. It was structured around aerobic activities, primarily walking, combined with a variety of tasks and playful exercises designed to enhance attention, coordination, postural control, balance, spatial and temporal structuring, interaction, listening, and empathy. The program was part of a broader project called “Brain-Gym,” funded by the Spanish State Research Agency. Brain-Gym aimed to evaluate a cognitive training intervention using a Brain-Computer Interface (BCI) system to stimulate specific neural circuits. Participants in the “Brain-Gym” project engaged in one individual BCI training session and one weekly group physical activity session for a duration of 12 weeks.

The “Cogn-Mover” sessions included a 10-min period for personal interaction and exchange before the 60-min main session, followed by 10–15 min for further interaction and farewell. These pre- and post-session phases enabled participants to engage with two of the study’s investigators, who also served as program facilitators. Conversations during these phases were often guided by physical activity journals maintained by participants, which documented their activity habits, physical sensations, and personal experiences. These exchanges inspired a deeper exploration of participants’ experiences through follow-up interviews.

All participants were Spanish and had grown up in socio-economic and cultural contexts where organized physical activity was not prioritized or widely promoted, especially in rural areas and urban neighborhoods with limited resources. Physical activity during their youth was typically associated with labor-intensive tasks, such as agricultural work, manual transportation, or walking, rather than structured exercise or sports. During their working years, responsibilities related to employment and family, combined with long hours and limited leisure opportunities, shaped their relationship with physical activity. Exercise was often regarded as a luxury, accessible primarily to younger individuals or those with ample free time. Furthermore, access to sports facilities and programs was scarce, particularly in less developed regions.

With retirement, participants experienced significant lifestyle changes. The cessation of work obligations provided time for self-care, while public health campaigns emphasizing the benefits of physical activity in preventing age-related conditions, such as cognitive decline and cardiovascular diseases, prompted a reevaluation of their habits and priorities.

The “Cogn-Mover” program consisted of weekly group sessions lasting 60 min over 12 weeks. These sessions were carefully designed to address both physical and cognitive health through practical and engaging activities, offering participants a holistic experience. The program employed a circuit-based format, incorporating exercises targeting joint mobility, balance, coordination, and functional strength. Activities were tailored to individual abilities, including gentle movements to improve flexibility, one-legged balance exercises, lateral movements, and tasks requiring reaction speed, such as responding to visual or auditory stimuli while performing specific movements. Cognitive engagement was integrated through dual-task activities, such as executing movements while processing cognitive stimuli, as well as group games aimed at stimulating memory and attention. The sessions concluded with stretching and relaxation exercises to promote well-being and reduce tension.

Beyond physical and cognitive health, the program was designed to foster community and social connections among older adults. It aligned with broader public health goals by promoting active aging and addressing the social isolation often experienced in this demographic. Furthermore, the program sought to challenge societal narratives of aging as a period of inevitable decline, instead highlighting the potential for growth and adaptation. The weekly sessions provided a dynamic, inclusive space where participants could rediscover their capabilities and engage meaningfully with others.

This holistic approach sought not only to improve physical condition but also to stimulate key cognitive processes, helping participants maintain active and healthy aging.

### 2.2 Data collection

Semi-structured interviews were conducted to allow participants to share their life stories concerning their bodies and physical activity. The interviews were transcribed verbatim and ranged from 50 to 75 min in duration.

Descriptive, emotional, sensory, experiential, and embodied data were collected (Lambert, 2020). Guiding questions were designed to evoke embodied and affective experiences related to movement throughout their lives. Examples of these questions included: What types of physical activity do you practice? Which ones do you enjoy the most? How does physical activity influence your perception of feeling strong or fragile? Is there any connection between physical activity and your sense of masculinity? What would you say about your body? Has your perception of your body changed over time? How has physical activity changed for you over the years? Are there situations in your daily life where you feel stronger or more vulnerable?

Since the study aims to construct narratives that position the body as a central interlocutor, it focuses on embodied and sensory experiences, reflecting on senses, emotions, responses, and triggers, as well as questions that encourage body-centered storytelling. The research explored how affect, sensitivity, and emotion emerged in these narratives and the capacities they generated or limited. Special attention was given to understanding the embodied energies present in the participants’ stories and conversations, as they were lived and felt “in the flesh” (Chadwick, 2017).

All participants provided informed consent prior to their participation in the study. To ensure confidentiality, pseudonyms were used in place of participants’ real names. The study has received favorable approval from the Research Ethics Committee for the implementation of the research Project [Ethical code PI 23-3325 NO HCUV].

### 2.3 Data analysis

A narrative thematic analysis was conducted to identify and examine patterns or themes within the stories shared by participants (Sparkes and Smith, 2014). This method integrates elements of narrative analysis with a thematic focus, capturing both the explicit content and the implicit dynamics of the narratives. To ensure rigor, two researchers independently reviewed and coded the interviews, identifying initial themes. These themes were then discussed collaboratively among the research team to refine and consolidate them into broader categories, ensuring they accurately reflected the participants’ narratives. This iterative process facilitated diverse perspectives and a comprehensive understanding of the data. Furthermore, the analysis allowed us to explore not only the main themes but also the affective and embodied depth underlying the participants’ narratives (Chadwick, 2017), valuing the complexity of individual experiences while identifying shared patterns that contribute to a holistic understanding of their lived experiences.

The following table (see Table 1) presents the categories derived from the narrative thematic analysis, along with the emerging themes associated with each category:

**Table 1:** Study categories and emerging themes associated with each.

Categories	Emerging themes
Physical activity and well-being	Importance of physical activity in daily life Perceived benefits of exercise on physical and mental well-being Preferences for outdoor activities versus indoor activities Impact of exercise on health perception and mobility
Perception of masculinity	Reflections on masculinity in the context of physical exercise Perceived differences in physical abilities between men and women Influence of gender stereotypes on participation in physical activities
Aging and the body	Bodily changes with age and their acceptance Feelings of fragility versus strength associated with aging Concerns about the Loss of mobility and abilities with aging Social stigma associated with aging
Social integration	Group dynamics in physical activities Barriers and motivations for participation in community activities Experiences of inclusion and exclusion in recreational spaces
Perception and attitudes toward exercise	Attitudes toward one’s own body and self-image Influence of exercise on self-esteem Differences in perceived physical well-being between men and women

## 3 Results and discussion

The relationship between physical exercise, masculinity, and aging is analyzed through the narratives of participants, exploring the dynamics of body, gender, and affect that emerge in their experiences. The results reveal a process of re-signifying the body and masculinity, while also uncovering the tensions and possibilities that physical movement offers in later life.

### 3.1 Physical activity and well-being

Participants consistently describe physical exercise as a cornerstone for maintaining vitality and a sense of purpose in daily life. Beyond the obvious physical benefits, exercise emerges as a deeply emotional and reflective practice. Carlos (70 years old) states: “When I go out for a run, it feels like everything else disappears for a while. It’s my time to be with myself, to leave everything else behind.” This narrative underscores how movement fosters emotional balance and serves as a coping mechanism for managing the psychological challenges of aging (Parra et al., 2019). Physical activity becomes not just a task, but a ritual—a moment of solitude and self-awareness that reconnects individuals with their bodies and minds.

Outdoor activities, in particular, hold special significance for participants, often described as a preferred context for exercise. Andrés (66 years old) shares: “I enjoy that more. Yesterday I went down to the valley, by the Esgueva River. Nature recharges your batteries.” This highlights the role of natural environments in amplifying the sensory and affective dimensions of physical activity. The act of moving outdoors intertwines bodily and spatial awareness, creating a deeper sense of connection to one’s surroundings. Phoenix and Sparkes (2008) emphasize that environments like these, act as catalysts for re-signifying aging, offering opportunities to redefine one’s relationship with the body and the self. Moreover, this aligns with biophilia theories (Tidball, 2012; Kellert and Wilson, 1993), which suggest that humans have an innate connection to nature, deeply rooted in our evolutionary past. This connection allows participants to find solace and revitalization in the natural world, transcending the physical aspects of exercise to tap into a profound sense of well-being.

Participants also emphasize how physical activity helps counteract feelings of vulnerability and loss of strength often associated with aging. Pablo (74 years old) reflects: “When I exercise, I feel like I’m getting stronger, as if that fragility you sometimes feel dissipates.” This sentiment encapsulates the transformative potential of movement, which not only reinforces the body but also redefines self-perception. Exercise becomes a means of reclaiming agency and autonomy in the face of physical decline, allowing participants to confront the aging process with resilience (Sparkes, 2015).

In addition, the narratives reveal how exercise is reframed as a source of pleasure rather than obligation. For example, Antonio (71 years old) explains: “When I walk outdoors, it’s not about how far I go or how fast. It’s about enjoying the moment and feeling good in my body.” This shift in focus—from achieving specific goals to experiencing movement for its intrinsic value—illustrates how participants embrace exercise as an act of celebration and self-care.

### 3.2 Perception of masculinity

The participants’ reflections on masculinity reveal a complex interplay between deconstruction of traditional gender norms and lingering societal expectations. Andrés (66 years old) observes: “Here at the gym, I see women lifting the same weights as men. It’s not like before; it does not matter who does what anymore.” This narrative reflects a shift in the perception of physical strength as an exclusively masculine trait, demonstrating how contemporary exercise spaces can challenge and dismantle traditional gender binaries (Tannenbaum and Frank, 2011).

However, tensions persist, particularly in how certain activities are culturally coded. Carlos (70 years old) states: “Sometimes I see other men and feel like they are still trapped in the idea that if you do something like yoga, you are no longer a real man.” This statement highlights the persistence of gendered stereotypes, which frame activities such as yoga as incompatible with traditional notions of masculinity. Such narratives underline the broader societal pressures older men face in reconciling their personal interests with societal expectations of gender roles.

Some participants, however, manage to transcend these stereotypes and redefine masculinity on their own terms. Uriel (73 years old) reflects: “I do Pilates, and I love it. At first, it felt strange, but then I realized that does not define who I am as a man. What matters is that I feel good.” This testimony illustrates the potential for a more fluid and inclusive understanding of masculinity, where physical activities traditionally considered feminine become spaces for self-expression and identity transformation. Merleau-Ponty’s (2012) concept of the body as a medium for experience resonates here, emphasizing how movement fosters a reimagining of one’s identity beyond rigid cultural constructs.

Participants also noted how engaging in diverse physical activities allowed them to redefine their notions of strength and capability. Andrés (66 years old) explained: “Strength is not just about lifting heavy weights anymore. It’s about endurance, flexibility, and feeling in control of my body.” This evolving definition of masculinity aligns with recent scholarship advocating for a more holistic view of gender identity in later life (Phoenix et al., 2005; Sparkes, 2015).

Additionally, the role of peer interaction in reshaping perceptions of masculinity cannot be overlooked. Group activities provided a context where traditional norms could be challenged collectively. Tomás (72 years old) shared: “Seeing other men my age trying yoga or Pilates made me realize it wasn’t just for women. It’s about health, not stereotypes.” This narrative highlights how social environments play a crucial role in enabling men to explore and embrace non-traditional activities, fostering a sense of solidarity and shared growth.

Participants’ reflections reveal an ongoing negotiation between societal expectations and personal fulfillment. Physical activity emerges as a space where masculinity is continually redefined, allowing men to break free from restrictive norms and embrace a more nuanced, adaptive understanding of their identities in later life.

### 3.3 Aging and the body

Participants in this study demonstrate a complex interplay of acceptance, adaptation, and resilience in their responses to the bodily changes associated with aging. Antonio (71 years old) reflects: *“Over the years, I’ve learned to listen to my body more. I know when I can push myself and when I need to stop.”* This statement underscores the importance of self-awareness and sensitivity toward the aging body, which acts not only as a biological entity but as an archive of memories and lessons accumulated over a lifetime (Niklasson et al., 2024). This approach to self-care emphasizes the evolving relationship individuals develop with their bodies, shifting from demands of peak performance to a more attuned understanding of bodily capabilities and limitations.

Despite this acceptance, the fear of losing physical capabilities remains a recurrent concern among participants. Carlos (70 years old) articulates this worry: “*I worry that one day I will not be able to move the same way. That’s why I try to stay active, even though I do not recover as quickly as I used to*.” His narrative reflects the tension between the desire to maintain an active lifestyle and the inevitable changes that accompany aging. Exercise, in this context, becomes a form of resistance against cultural narratives that equate aging with decline. Instead, it offers a proactive way to project future possibilities for the body and to remain engaged with life’s physical demands (Young et al., 2024).

Participants frequently express that aging has prompted a reevaluation of their relationship with their bodies, resulting in a more flexible and realistic approach to physical activity. Andrés (72 years old) captures this perspective, stating: “*I know I cannot do the same things I used to, but that does not mean I’m going to stop trying. My body still has a lot to offer*.” His optimism contrasts sharply with deficit-based discourses on aging, which often portray the aging body as solely a site of loss and fragility. Instead, Andrés reframes his limitations as opportunities for growth, self-discovery, and adaptation. This sentiment aligns with Dionigi et al. (2013), who emphasize that aging bodies are dynamic and capable of ongoing reinvention.

The narratives reveal that exercise is not merely a physical activity but also a symbolic act of agency and control. For many participants, movement represents an opportunity to push back against feelings of vulnerability and to assert autonomy over their aging bodies. Antonio (71 years) describes his routines as a way to “*stay in control of what I can, even when my body changes*.” This perspective highlights the capacity of physical activity to reinforce self-efficacy and mitigate anxieties about the aging process, turning exercise into a transformative practice that strengthens both body and mind.

Furthermore, participants underscore the role of bodily awareness in navigating aging. This awareness is not static but evolves over time, as older adults learn to interpret and respond to the signals their bodies provide. The process involves reconciling past identities—shaped by youthful vigor and capability—with current realities that require adaptation. As Antonio (71 years) notes, this adjustment is not about resignation but about learning: “*I’ve learned to appreciate what my body can still do, even if it’s different from before*.” This notion of the body as a site of continuous learning reflects Garrett’s (2024) exploration of embodied adaptation, where individuals align their practices with their lived experiences, creating spaces of resilience and agency.

The role of movement extends beyond physical benefits; it becomes a source of emotional and existential renewal. Participants frequently describe exercise as a way to reconnect with their bodies and experience vitality. Pablo (74 years old) states: “*When I exercise, it reminds me that I’m still here, still capable*.” This connection reinforces the idea that bodies in motion are not just physical entities but also bearers of identity and purpose. Exercise helps participants resist the cultural tendency to marginalize older bodies, affirming their value and potential in a society that often prioritizes youth and productivity.

In addition, the study reveals how physical activity fosters a balance between acceptance and aspiration. While participants acknowledge that their bodies may not function as they once did, they remain committed to maintaining an active lifestyle. This balance reflects a broader shift from performance-driven goals to holistic engagement with movement. Andrés summarizes this shift: “*It’s not about doing more or being better than anyone. It’s about doing what feels right for me*.” This perspective echoes Phoenix and Sparkes’ (2008) findings that older adults often redefine physical activity as a personal and meaningful practice rather than a competitive or performance-based one.

Ultimately, the participants’ experiences illuminate the transformative potential of aging bodies. Exercise emerges not only as a strategy for maintaining health but also as a profound act of self-expression, resilience, and empowerment. These narratives challenge deficit-based views of aging, instead portraying it as a dynamic process where the body remains a site of possibility and renewal. By fostering autonomy, strengthening self-esteem, and providing avenues for self-discovery, physical activity becomes an essential tool for navigating the challenges and opportunities of aging.

### 3.4 Social integration

Group physical activities emerge as pivotal spaces for fostering inclusion, social connection, and a sense of belonging among older adults. José (68 years old) reflects: “*In the hiking group, aside from exercising, we share many things. I no longer feel as isolated as I did when I retired*.” This statement underscores how group dynamics can effectively counteract social isolation, a common issue in older adulthood, while providing a framework for building emotional and social bonds (Klinenberg, 2018; Mulcahy, 2012). The act of engaging in shared physical activities offers participants not only an opportunity for movement but also a platform for creating meaningful relationships that extend beyond the exercise itself.

Participants also emphasized how shared activities provided a sense of routine and purpose. Andrés (66 years old) explained: “*Having a set day for hiking gives structure to my week. I wake up knowing I’ll see the group and spend time outdoors—it’s something I look forward to*.” This testimony highlights how the predictability and regularity of group activities can help combat the aimlessness that some retirees report feeling after leaving the workforce.

Moreover, inclusive spaces can break down initial discomforts related to gender, age, or perceived physical abilities. Tomás (72 years old) shared: “*At first, I felt uncomfortable in the fitness classes because I did not know anyone, and I thought everyone else was more experienced. But now, I feel valued and welcomed*.” Such narratives reflect how environments that prioritize inclusion and mutual respect can create opportunities for learning, growth, and mutual support (Halaweh et al., 2016).

The social integration achieved through group activities also has a spillover effect into other aspects of participants’ lives. For example, Antonio (71 years old) mentioned: “*Since joining the walking group, I’ve become more confident talking to others. I’ve even reconnected with old friends because I feel more comfortable putting myself out there*.” These accounts highlight the transformative potential of group physical activities, not only for enhancing physical and emotional well-being but also for reinforcing community ties and enriching participants’ social lives.

### 3.5 Perceptions and attitudes toward exercise

Participants consistently described exercise as a practice deeply intertwined with self-care, self-awareness, and personal growth. Antonio (71 years old) remarked: “*Exercise is my meditation time. It’s when I can truly feel my body and understand what it needs*.” This view reflects the role of physical activity as a tool for reconnecting with one’s body, fostering mindfulness, and managing the demands of aging. Movement becomes a medium through which participants can navigate the physical and emotional challenges associated with growing older, transforming their relationship with their bodies (Dionigi et al., 2013; Hickey and Austin, 2007).

A recurring theme in participants’ narratives was the shift from viewing exercise as an obligation to embracing it as a source of joy and vitality. Uriel (73 years old) shared: “*Even though my body has changed, I still see exercise as something positive. I do not do it out of obligation but because it makes me feel alive*.” This perspective underscores how physical activity becomes an act of resistance against the narratives of decline that often accompany aging. Rather than being framed solely as a means of maintenance, exercise emerges as a celebration of bodily capabilities and a source of empowerment (Clarke et al., 2020; Bredland et al., 2018; Phoenix et al., 2005; Phoenix and Sparkes, 2008).

The participants also highlighted the flexibility and adaptability of their exercise routines. Andrés (72 years old) explained: “*I used to think exercise had to be intense to matter. Now, I realize that even gentle movements, like stretching or walking, can make a big difference in how I feel*.” This shift demonstrates how perceptions of exercise evolve over time, moving from performance-driven goals to an appreciation of its holistic benefits for body and mind.

Several participants noted that physical activity helped them reframe their self-image in the context of aging. Carlos (70 years old) commented: “*When I exercise, I feel strong. It’s a reminder that my body is still capable, even if it’s different from when I was younger*.” Such reflections highlight how exercise can reinforce a positive self-perception, countering feelings of fragility and fostering resilience.

Additionally, some participants described physical activity as a gateway to rediscovering the pleasures of movement. Pablo (74 years old) shared: “*I never thought I’d enjoy yoga, but it’s become one of my favorite things. It’s not about pushing myself anymore—it’s about moving in a way that feels good*.” This statement exemplifies the transformative potential of exercise, where participants redefine their relationship with their bodies, focusing on well-being rather than external expectations or comparisons.

The narratives also revealed how physical activity fosters a sense of autonomy. Uriel (73 years old) explained: “*Exercise reminds me that I have control over my body, even as I age. It’s something I do for myself, on my own terms*.” This empowerment challenges deficit-based discourses surrounding aging, framing movement as a tool for maintaining independence and agency (Phoenix and Sparkes, 2008; Sparkes, 2015).

Through these reflections, it becomes evident that exercise transcends its physical benefits, emerging as a transformative practice that shapes attitudes, reinforces identity, and fosters resilience in the face of aging. The narratives illustrate how movement serves as both a celebration of capability and a vehicle for reimagining one’s relationship with the body, self, and the aging process.

## 4 Conclusion

The findings of this study highlight the central role of physical exercise as a space for the renegotiation of masculine identities and the embodied experiences of older men. Through narrative analysis, participants reveal how they challenge and transform traditional notions of masculinity, moving away from stereotypes focused solely on physical strength and endurance. Instead, they adopt practices emphasizing self-care, holistic health, and emotional well-being, reflecting a broader cultural shift toward more inclusive understandings of masculinity. These findings align with Gough and Robertson’s (2010) critique of hegemonic masculinity and Garrett’s (2004) insights on the social construction and potential transformation of gendered bodies. Exercise becomes a medium for reconfiguring relationships with their bodies and societal expectations, supporting a dynamic interplay between identity and movement.

Physical activity also emerges as a means of rethinking aging as a stage of possibilities and adaptations rather than an inevitable process of loss. While participants acknowledge the physical limitations that accompany aging, they emphasize how these constraints have fostered greater bodily awareness and reconnection with their physical selves. Niklasson et al. (2024) discuss the alignment of physical activity with lived experiences, which is evident in how participants perceive exercise as a space for resilience, autonomy, and self-esteem. Movement helps address fears associated with aging, such as fragility and the loss of mobility, enabling participants to confront these challenges with optimism and adaptability (Young et al., 2024).

The emotional and affective dimensions of exercise also play a central role in participants’ experiences. Exercise not only impacts their physical well-being but also revitalizes their sense of identity, purpose, and emotional connection. Carlos’s narrative about running as a form of solitude and self-reconnection echoes Parra et al.’s (2019) findings on the emotional balance fostered by movement. Similarly, Andrés’s preference for outdoor activities highlights how natural environments amplify embodied experiences, offering a deeper connection to oneself and the surroundings. These insights align with biophilia theories (Tidball, 2012; Kellert and Wilson, 1993), which emphasize the inherent human connection to nature as a source of well-being.

The narratives further reveal a process of deconstructing and renegotiating masculinity. Participants reflect on their changing perceptions of traditional masculine ideals, as seen in Andrés’s observation about women lifting weights, which challenges the exclusivity of physical strength as a male attribute (Tannenbaum and Frank, 2011). However, tensions persist, particularly in activities like yoga, which some participants associate with a perceived loss of masculinity. These findings underscore the complexity of navigating masculinity in later life, where traditional norms intersect with evolving personal and social expectations. Nonetheless, narratives like Uriel’s embrace of Pilates demonstrate how older men redefine masculinity in fluid and adaptive ways, aligning with Merleau-Ponty’s (2012) perspective on the body as a site of transformation and identity renegotiation.

Social integration emerges as another significant dimension of physical exercise. Group activities are described as spaces that promote inclusion, combat social isolation, and foster meaningful interactions. José’s narrative about the hiking group echoes Klinenberg’s (2018) discussion of social infrastructure as a tool for combating loneliness and fostering community ties. These group settings also provide opportunities to overcome initial discomfort, as noted by Tomás, who eventually found a sense of belonging and support in his fitness class. These findings align with Halaweh et al.’s (2016) emphasis on the role of habitual physical activity in maintaining social roles and fostering inclusion. Furthermore, group dynamics create environments where participants can share experiences and develop emotional bonds, as noted by Mulcahy (2012) in discussions on affective assemblages.

This study broadens perspectives on active aging by emphasizing the interaction between moving bodies, notions of masculinity, and the affective and social dynamics shaping these experiences. It demonstrates how aging bodies resist deficit-based narratives, instead serving as spaces of possibility and transformation (Clarke et al., 2020; Bredland et al., 2018; Phoenix and Sparkes, 2008; Sparkes, 2015). Given the growing prominence of demographic aging, it is crucial to continue exploring the intersections of aging, gender, and physical activity across diverse cultural and social contexts. The findings of this study have important implications for health policy and practice. To promote active aging among older men, public health interventions should prioritize creating inclusive physical activity programs that address economic and geographical barriers. Additionally, campaigns should challenge traditional gender norms by emphasizing the holistic benefits of exercise, including emotional well-being and social integration. Policymakers should also invest in infrastructure, such as accessible sports facilities and outdoor spaces, to encourage participation across diverse communities.

Future research could compare the experiences of older men and women in exercise, considering variables such as social class, rural–urban divides, and generational differences. Furthermore, public policies and physical activity programs must foster inclusive and accessible environments to enhance the capacities and opportunities of older adults. As Garrett (2024) emphasizes, these efforts can provide a nuanced and positive understanding of aging, not as a decline but as a dynamic process filled with possibilities.

## Data Availability

The original contributions presented in the study are included in the article/supplementary material, further inquiries can be directed to the corresponding author.
